# Supplementary material for: Hot spot prediction in protein-protein interactions by an ensemble system
Source: BMC Syst Biol. 2018 Dec 31;12(Suppl 9):132. doi: 10.1186/s12918-018-0665-8 (PMC6311905; doi:10.1186/s12918-018-0665-8)
Supplement: Supplementary file 2 — All relevant MATLAB code. A simple Matlab implement of our predictor. (ZIP 19,742 kb) [file 12918_2018_665_MOESM2_ESM.zip › Additional file 2/Read ASA/ASA Property/SKEMPI/1GCQC.txt]

 NetSurfP - Protein Surface Accessibility and Secondary Structure Predictions

  

|  |  |  |
| --- | --- | --- |
|  |  | NetSurfP - Protein Surface Accessibility and Secondary Structure Predictions  Technical University of Denmark |

  


---

```
# For publication of results, please cite:
# A generic method for assignment of reliability scores applied to solvent accessibility predictions.
# Bent Petersen, Thomas Nordahl Petersen, Pernille Andersen, Morten Nielsen and Claus Lundegaard
# BMC Structural Biology 2009, 9:51 doi:10.1186/1472-6807-9-51
#
# Column 1: Class assignment - B for buried or E for Exposed - Threshold: 25% exposure, but not based on RSA
# Column 2: Amino acid
# Column 3: Sequence name
# Column 4: Amino acid number
# Column 5: Relative Surface Accessibility - RSA
# Column 6: Absolute Surface Accessibility
# Column 7: Z-fit score for RSA prediction
# Column 8: Probability for Alpha-Helix
# Column 9: Probability for Beta-strand
# Column 10: Probability for Coil
E G  1GCQC                  1    0.898  70.665  -1.441   0.003   0.003   0.994
E S  1GCQC                  2    0.654  76.696  -1.319   0.018   0.019   0.964
E H  1GCQC                  3    0.501  91.150  -1.185   0.018   0.019   0.964
B M  1GCQC                  4    0.207  41.421   0.017   0.058   0.017   0.925
B P  1GCQC                  5    0.244  34.567  -0.709   0.115   0.016   0.868
E K  1GCQC                  6    0.332  68.375   0.882   0.307   0.165   0.527
B M  1GCQC                  7    0.082  16.468  -0.056   0.252   0.423   0.325
B E  1GCQC                  8    0.167  29.245   0.661   0.172   0.690   0.138
B V  1GCQC                  9    0.039   6.025  -0.323   0.172   0.690   0.138
B F  1GCQC                 10    0.022   4.395   1.061   0.273   0.587   0.140
B Q  1GCQC                 11    0.134  23.843   0.466   0.373   0.401   0.226
B E  1GCQC                 12    0.257  44.968  -0.732   0.321   0.252   0.427
B Y  1GCQC                 13    0.137  29.234   0.139   0.191   0.086   0.723
B Y  1GCQC                 14    0.266  56.759  -0.845   0.113   0.043   0.844
E G  1GCQC                 15    0.360  28.293  -2.065   0.018   0.047   0.935
B I  1GCQC                 16    0.222  41.089  -0.788   0.005   0.015   0.979
B P  1GCQC                 17    0.216  30.665  -1.157   0.005   0.015   0.979
E P  1GCQC                 18    0.388  55.015  -0.826   0.005   0.015   0.979
B P  1GCQC                 19    0.258  36.596  -2.084   0.018   0.019   0.964
E P  1GCQC                 20    0.369  52.304  -1.695   0.018   0.019   0.964
B G  1GCQC                 21    0.214  16.850  -2.222   0.018   0.019   0.964
E A  1GCQC                 22    0.513  56.577  -1.819   0.018   0.019   0.964
E F  1GCQC                 23    0.408  81.805  -1.740   0.018   0.019   0.964
B G  1GCQC                 24    0.178  14.024  -1.712   0.018   0.019   0.964
B P  1GCQC                 25    0.203  28.735  -0.407   0.018   0.019   0.964
B F  1GCQC                 26    0.105  20.993  -1.107   0.020   0.205   0.775
B L  1GCQC                 27    0.042   7.635  -0.301   0.021   0.451   0.528
E R  1GCQC                 28    0.256  58.510   0.610   0.022   0.552   0.426
B L  1GCQC                 29    0.036   6.628  -0.440   0.022   0.359   0.619
E N  1GCQC                 30    0.355  51.943   0.127   0.020   0.205   0.775
E P  1GCQC                 31    0.396  56.249  -0.199   0.018   0.088   0.893
E G  1GCQC                 32    0.262  20.612  -1.201   0.053   0.043   0.903
B D  1GCQC                 33    0.209  30.074  -0.475   0.052   0.084   0.864
B I  1GCQC                 34    0.077  14.190   0.062   0.085   0.775   0.140
B V  1GCQC                 35    0.050   7.746   0.612   0.018   0.846   0.136
B E  1GCQC                 36    0.132  23.078   0.601   0.018   0.846   0.136
B L  1GCQC                 37    0.035   6.372   0.174   0.018   0.846   0.136
B T  1GCQC                 38    0.066   9.099   0.308   0.023   0.655   0.322
E K  1GCQC                 39    0.290  59.550   0.438   0.021   0.451   0.528
B A  1GCQC                 40    0.109  11.957  -1.262   0.021   0.279   0.699
E E  1GCQC                 41    0.324  56.533  -0.055   0.018   0.088   0.893
B A  1GCQC                 42    0.170  18.756  -1.185   0.052   0.084   0.864
E E  1GCQC                 43    0.373  65.076  -0.516   0.052   0.084   0.864
B H  1GCQC                 44    0.189  34.343  -0.314   0.113   0.087   0.800
B N  1GCQC                 45    0.239  34.975  -0.041   0.113   0.087   0.800
B W  1GCQC                 46    0.105  25.277  -0.549   0.125   0.227   0.648
B W  1GCQC                 47    0.084  20.178  -0.636   0.135   0.317   0.548
E E  1GCQC                 48    0.293  51.170   0.142   0.135   0.317   0.548
B G  1GCQC                 49    0.119   9.389  -1.952   0.135   0.317   0.548
E R  1GCQC                 50    0.295  67.601   0.322   0.069   0.386   0.545
B N  1GCQC                 51    0.245  35.912  -1.105   0.069   0.386   0.545
B T  1GCQC                 52    0.119  16.533   0.230   0.066   0.296   0.638
B A  1GCQC                 53    0.206  22.701  -1.060   0.066   0.296   0.638
E T  1GCQC                 54    0.276  38.240  -1.084   0.064   0.216   0.721
E N  1GCQC                 55    0.470  68.764  -1.088   0.019   0.141   0.840
E E  1GCQC                 56    0.396  69.216  -0.610   0.020   0.205   0.775
B V  1GCQC                 57    0.133  20.504  -0.304   0.022   0.359   0.619
B G  1GCQC                 58    0.104   8.161  -2.696   0.022   0.359   0.619
B W  1GCQC                 59    0.123  29.509  -0.418   0.022   0.359   0.619
B F  1GCQC                 60    0.078  15.715   0.363   0.021   0.279   0.699
B P  1GCQC                 61    0.131  18.617  -0.186   0.020   0.205   0.775
B C  1GCQC                 62    0.187  26.241  -1.134   0.018   0.047   0.935
E N  1GCQC                 63    0.496  72.658  -1.204   0.018   0.047   0.935
E R  1GCQC                 64    0.355  81.364  -0.248   0.018   0.088   0.893
B V  1GCQC                 65    0.149  22.871  -1.024   0.020   0.205   0.775
E H  1GCQC                 66    0.429  77.944   0.642   0.020   0.205   0.775
E P  1GCQC                 67    0.395  56.065  -1.351   0.019   0.141   0.840
E Y  1GCQC                 68    0.325  69.538  -0.378   0.018   0.088   0.893
E V  1GCQC                 69    0.661 101.657  -1.647   0.003   0.003   0.994

Explain the output.  Go back.
```

---
